# Postnatal home visits by health extension workers in rural areas of Ethiopia: a cross-sectional study design

**DOI:** 10.1186/s12884-020-03003-w

**Published:** 2020-05-19

**Authors:** Yemane Berhane Tesfau, Alemayehu Bayray Kahsay, Tesfay Gebregzabher Gebrehiwot, Araya Abrha Medhanyie, Hagos Godefay

**Affiliations:** 1 College of Medicine and Health Sciences, Adigrat University, Adigrat, Ethiopia; 2 Mekelle University, College of Health Sciences, School of Public Health, Mekelle, Ethiopia; 3 Tigray Regional Health Bureau, Mekelle, Ethiopia

**Keywords:** Postnatal home visits, Health extension workers, Rural districts, Northern Ethiopia

## Abstract

**Background:**

In low-income countries like Ethiopia, where families have poor access to or do not utilize the services of formal health care systems, community health workers provide postnatal care services through home visits. However, the extent and effectiveness of home-based postnatal visits by community health workers such as the Ethiopian health extension workers (HEWs) are not well explored. This community -based study aimed to determine the coverage, contents of postnatal home visits and associated factors by health extension workers in Northern Ethiopia.

**Methods:**

We conducted a community based cross-sectional study in the rural Districts in Northern Ethiopia from August to September 2018. A total of 705 mothers who gave a live birth in the year preceding the survey were selected using multistage random sampling. A structured questionnaire was applied to collect data by interviewing the mothers. Data were analyzed using SPSS version 22 statistical software. Association of postnatal home visits with possible explanatory variables was investigated using logistic regression.

**Results:**

One hundred and two (14.5%) mothers and newborns received PNC home visit within three days after birth from HEW and 170(24.1%) reported postnatal home visits within 42 days. Among the mothers who received postnatal home visits, 6.5% measured their blood pressure, 11.2% measured their temperature, 20% counseled about family planning, 16.5% counseled on newborn danger signs, 11.2% counseled on the skin to skincare of the newborn and 14.1% of their newborns were measured their weight at home. Mothers who received at least one home visit during pregnancy (AOR, 7.49; CI 3.55–15.80), participated in pregnant women forum (AOR, 3.16; CI 1.67–5.99), notified their birth (AOR, 6.16; CI 3.50–10.84) and those members of community health insurance (AOR, 1.87; CI 1.13–3.10) were factors associated with postnatal home visit by a health extension worker.

**Conclusion:**

The coverage of postnatal home visits by health extension workers remains low in rural districts of Northern Ethiopia. The existing health systems should consider interventions that improve pregnancy and birth notification strategies and more efforts should be made at improving community-based participation and linkages with community health workers.

## Background

Despite the remarkable achievements observed, about 4 million neonates and 287,000 maternal deaths occur each year, mostly in low- income countries, due to maternal and newborn complications occurring within 24 h of birth and thereafter [1]. In Ethiopia, maternal and neonatal mortality remains the highest among the world, at 412/100,000 and 30/1000, respectively. The trends from the previous surveys in Ethiopia showed that a continuous decline in infant and under − 5 mortality preceding each respective survey, however, the trend among the neonatal mortality decreased from 39 to 29 between the 2005 and 2016 EDHS, but has remained stable since the 2016 EDHS [2].

Despite the fact improvements observed with antenatal care (ANC) and facility delivery, postnatal care (PNC) utilization coverage remains low due to many reasons such as unavailability, inaccessibility, poor quality of health services, socio-cultural beliefs, awareness on danger signs of postnatal period, and distance [3–10]. Even with different multiple interventions, postnatal maternal and newborn care utilization health facility remains low [2, 11]. Studies also showed that a significant proportion of mothers prefer to return home or discharged within a few hours after delivery, which makes them not receive the required care [12, 13].

Studies in low and middle -income countries with high new-born mortality demonstrated that early postnatal home visits, by community health workers help to reduce neonatal deaths and improve maternal and neonatal health. For example, studies conducted in India, Bangladesh, and Pakistan have shown that home visits can reduce deaths of newborns by 30–61% in developing countries where there is a high mortality. In particular, home visits improved coverage of the key high-impact and cost-effective neonatal interventions such as early initiation of breastfeeding, skin-to-skin contact between newborns and their mothers, delayed bathing of the newborn, hygienic care of the baby’s umbilical cord stump [14].

Based on the experiences and evidence from South Asian trials, in 2009, World Health Organization (WHO) and United Nations Children’s Fund (UNICEF) issued a joint statement recommending postnatal home visits (PNHVs) for delivery of postnatal care. Following the 2009 Joint Statement, many countries adopted policies to deliver postnatal home visits. Among the 75 countries included in the *Countdown to 2015* report, 59 have policies deliver such home visits within 1 week of birth [15, 16].

The health extension program, which was launched in 2003 contributed to mobilizing community members towards the utilization of antenatal care and institutional delivery. HEWs were trained to give basic maternal and child health care and improve the health of the mothers and newborns during the antenatal, delivery and postnatal period and HEWs are expected to spend 75% of their time in the community and provide essential health services through the house to house visit [17].

Routine home visiting by community health workers during the postnatal period considers identification, assessment, management, and referral of both mother and baby for care. Postnatal care services provided at home for the mother include assessing general condition, checking vital signs and danger signs. For the newborns, it includes a general body examination, checking general danger signs, checking the cord for any bleeding and infection, and assessing breast feeding [18].

Studies on the coverage of the postnatal visit within 48 h showed low [19] and the coverage within 3 days after delivery from three Countries (Bangladesh, Nepal, and Malawi) by Community Health Workers (CHWs) showed that 57, 50, and 11% respectively, and the pooled results of the study in these countries found that early visits were more likely if a mother had been visited by CHWs during pregnancy, birth notification by CHWs, and home deliveries [20].

In Ethiopia, since the implementation of the health extension program (HEP), few studies have published findings on the coverage of PNHVs by HEWs. In Southern Ethiopia, 12.4% of mothers and their neonates were visited by the HEWs during the first month of birth and the major factors associated with early PNHVs were HEWs visit home during pregnancy, skilled delivery, and having HEW’s cell phone and no association were observed with maternal socio-demographic characteristics and early PNHVs [21].

In the northern Ethiopia, to improve the lives of mothers and newborns, different activities have conducted by HEWs in line with the government’s policies and strategies and EDHS report showed the percentage of facility-based postnatal care utilization within the first 2 days after delivery was 45.4% [22]. However, paucity exists on the evidence about the coverage of early PNHVs by HEWs. Moreover, little is known about the contents of the care given during PNHVs. Therefore, the study aimed to determine the coverage and contents of PNHVs and associated factors by HEWs, in rural Tigray, northern Ethiopia.

## Methods

### Study setting

The study was conducted in four districts of the South Eastern Zone of Tigray region, namely, Dogua Tembien, Enderta, Hintalo-Wajerat, and Seharti-Samre. This zone surrounds the capital city of Tigray region, Mekelle. The four districts had a total population of 567,735 with the total households estimated at 129,031. The zone has a total of 88 villages/kebeles (17-Enderta, 24- Dogua-Tembien, 23- Seharti-Samre and 24- Hintalo-Wajerat district). The estimated live births with children aged 11 months and below in the study area were 17,600. In 2018, there were 03 primary hospitals, 24 health centers and 75 health posts in the study zone. Concerning the number of health professionals, there were 731 health care providers in the zone out of which 183 were health extension workers (HEWs) [23].

### Study design and population

We conducted a community-based cross-sectional survey among mothers who gave live birth in the past year before the survey/data collection.

### Sample size and sampling technique

A single population proportion formula was used to determine the sample size. To consider the design effect of 1.7, we assumed a rate of homogeneity (Roh = 0.05) and Coefficient of variance (CV = 0.25) for unequal size clusters. Using a 10% of none response rate the total sample size was 705. The study employed a multi-stage sampling at the kebele and household level. All districts in the zone were involved in the study. In the first stage, 30 clusters/kebeles were selected using simple random sampling out of 88 clusters found in the zone (6-Enderta woreda, 8- Dogua-Tembien, 8- Seharti-Samre and 8- Hintalo-Wajerat). The list of mothers with infants less than 1 year was registered in each cluster. A total of 138 participants from Enderta, 178 participants from Dogua Tembien, 195 participants from Seharti-Samre and 194 participants from Hintalo-Wajerat district were recruited.

### Data source and measurement

Data were collected with an interviewer-administered structured questionnaire that was adapted from Ethiopia demography health survey (EDHS) and the last 10 km (L10K) survey [22, 24]. The tool contains items regarding socio-demographic, status towards model household, community based participations like pregnant women forum, women development group (WDG), and community health insurance membership, availability of HEW’s cell phone at home, time taken to visit the household, ANC attendance (both facility and home), place of delivery, birth notification, attendants at birth, postnatal visits and contents of PNC provided. It was initially prepared in English and then translated into the local language (Tigrigna) and translated back to English by different language experts. The questionnaire was pre-tested prior to the commencement of actual data collection outside the study districts. A total of 20 field workers (BSc and above in nursing, and midwifery) were recruited for the data collection and 2 days training was given for the data collectors. The interviewers visited each eligible woman at her home to administer the survey. Day to day supervision of the data collection was made by the principal investigator and supervisors of the study. A protocol that guides the design, implementation and management of the survey was developed and given to the data collectors. The supervisors reviewed all completed questionnaires in the field for accuracy, consistency, and completeness. Data were collected from August – September 2018. In this study, the postnatal home visit is defined at least one home visit by HEW within 3 days after childbirth. The coverage of postnatal home visits was defined as the percentage of women and/or newborns that were visited at home within 3 days after delivery. The contents of the postnatal home visits were measured by mothers/caretakers words.i.e.by mention or not mention technique (mention = 1, not mention = 0).

### Data analysis

The data were coded, entered, edited, and analyzed using SPSS Version 22.0. Descriptive statistics like: proportion, mean, and standard deviation were used and the results were presented using text, tables, and figures. Binary logistic regression was used to check the associations between explanatory variables and postnatal home visit. Multi co-linearity was checked by using Variance inflation factor and model fitness using Hosmer and Lemeshow Test was conducted prior to running to multivariable analysis. A *p*-value of less than 0.05 was considered to be statistically significant.

### Ethics approval and consent to participate

Ethical approval was obtained from the Institutional Review Board of Mekelle University (No.1437/2018). Verbal consent was also obtained from the study participants. We secured verbal consent as a significant number of the study subjects were unable to read and write. The use of verbal consent was approved by the ethics committee.

## Result

### Participant characteristics

We enrolled a total of 705 mothers with a 100% response rate in the study; of these, about 250(35.5%) were in the age group of 30 to 39 years with the mean age of 27.5 years (±5.77). About three-fourth, 502(71.2%) of the study participants can read and write a simple sentence, though, 284(40.3%) mothers had never attended a school. Almost all 701(99.4%) participants were Christian Orthodox. More than half 418(59.3%) of mothers were visited during pregnancy by a HEW. However, 371(88.8%) of the mothers did not receive visit during their first trimester. About 684(97%) of respondents reported making at least one antenatal care visit to a health facility, and 247(36.1%) reported 4 or more visits. Above half of the respondents, 390(57%) initiated antenatal care before 16 weeks of pregnancy. Facility deliveries were reported by 629(89.2%) of the participants, however, 591(81.8%) of them had a facility stay of less than 24 h post-delivery. The average number of live births per mother was 3.12(±1.9). Concerning to the distance from the health post majority of the mothers, 501(71.1%) found less than 30 min of walking distance (Table 1).

**Table 1:** Sociodemographic characteristics of mothers and their neonates in the rural Tigray region, Northern Ethiopia, 2018

Variables	Frequency	Percentage
Current maternal age		
18–24	245	34.8
25–29	183	26.0
30–39	250	35.4
40–49	27	3.8
Maternal education		
No education	284	40.3
Primary education	250	35.5
Secondary education and above	171	24.2
Marital status		
Married and/ live together	647	91.8
Divorced/separated/widowed	58	8.2
Sex of newborn		
Male	357	50.6
Female	348	49.4
Children ever born		
1	163	23.1
2	167	23.7
3	113	16
+ 4	262	37.2
Distance to nearest health facility (one way)		
< 30 min	501	71.1
30 min to < 1 h.	160	22.7
> =1 h	44	6.2

### Postnatal home visit coverage

**Fig. 1:**
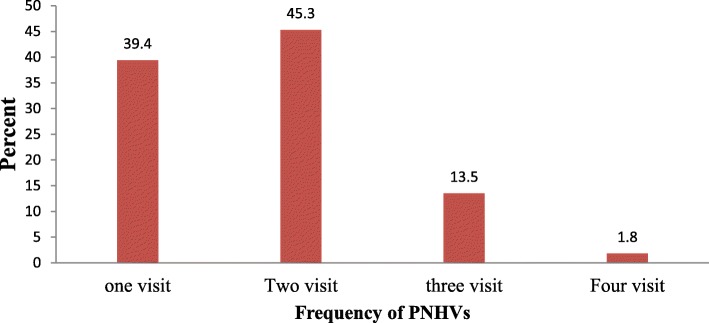
Proportion of mothers who gave birth by the number of visit received in Rural Tigray, northern Ethiopia

Overall, 102(14.5%) with (95% CI = 12.0 to 17.3) of mothers and their newborns received a home visit from a HEW within 3 days after birth. Within the Postnatal period, about 273 PNHVs and 170(24.1%) participants were visited by the HEWs. Out of the PNHVs performed, 67(39.4%) of mothers and their neonate received only one visit, 77(45.3%) received two visits, 23(13.5%) visited three times, and 03(1.8%) received four times by the HEWs (Fig. 1). Out of the total of home deliveries, only 08(10.5%) of mothers and their newborns received PNHVs within 3 days after delivery. Table 2 shows the timing of each visit where only 39(22.9%) mothers and their newborns received PNHV within the critical time i.e. within 24 h. In the second visit, the majority of the participants 71(92.2%) received PNHVs after the third day of delivery. In the third visit 18(78.3%) of mothers and their neonates received PNHVs after 7 days. Six out of 77 participants (7.8%) of their second visit were at 2–3 days and 5/23(21.7%) of the visit were at 4–7 days (Table 2).

**Table 2:** Coverage of PNHVs by HEWs in rural Tigray Northern Ethiopia, 2018

Maternal and newborn postnatal home receipt coverage(*N* = 705)	Frequency	%
Overall PNHVs within 42 days after delivery		
No	525	75.9
Yes	170	24.1
PNHV receipt within three days after delivery (n = 705)		
No	603	85.5
Yes	102	14.5
Initial PNHVs (*n* = 170)		
Within 24 h	39	22.9
2–3 days	63	37.1
4–7 days	53	31.2
After 7 days	15	8.8
Second PNHVs (*n* = 77)		
2–3 days	06	7.8
4–7 days	36	46.8
After 7 days	35	45.4
Third PNHVs (*n* = 23)		
4–7 days	05	21.7
After 7 days	18	78.3
Fourth PNHVs (*n* = 3)		
After 7 days	03	100

**Fig. 2:**
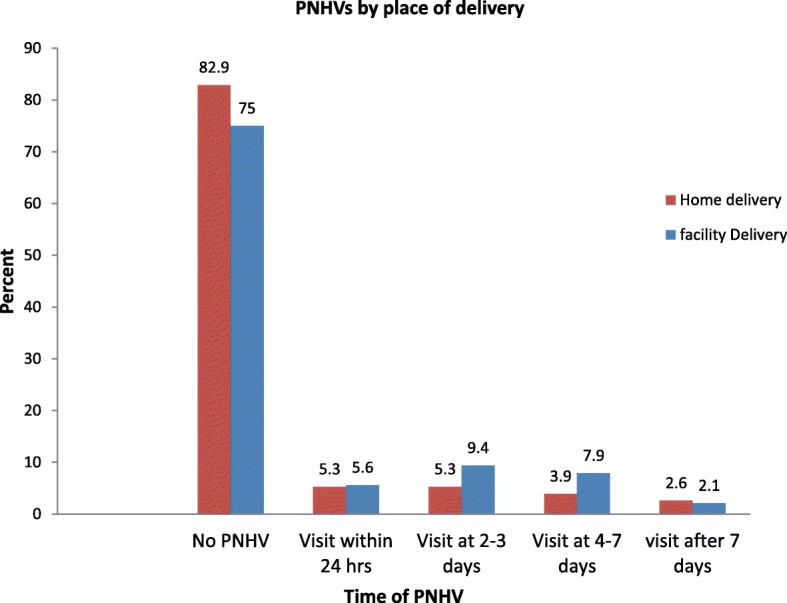
Percentage of mothers who received PNHVs within 42 days after birth by place of delivery

Out of 76 home deliveries, only 13(17.1%) and 08(10.5%) of mothers and their newborn received PNHVs in the postnatal period and within 3 days after delivery respectively. Whereas, 472(75%) of facility deliveries did not receive PNHVs after delivery. Immediately after delivery, 55(7.8%) and 31(4.4%) of mothers were visited by women development group (WDG) leaders and Traditional birth attendants respectively (Fig. 2**).**

**Fig. 3:**
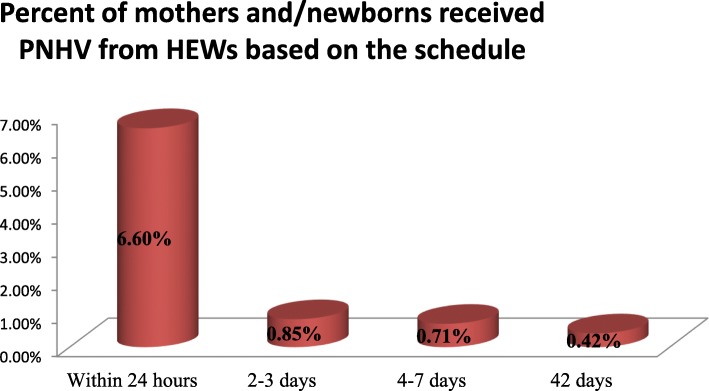
Percentage of mothers and newborns who received PNHVs from HEWs based on the schedule

Out of the total facility deliveries, 114 *(*18.2%) received initial postnatal care at *the facility* within 24 h. Because of this initial PNHV at home within 24 h was calculated out of 591 delivered mothers and it was 39 *(*6.6%). Six *(*0.85%) out of 705 mothers received PNHV their second visit *in* 2–3 days; 5/0.71%) mothers and their newborns received their third visit *in* 4–7 days and only 03/0.42%) mothers received their fourth visit at 42 days (Fig. 3).

### Postnatal care contents provided during a home visit

Out of 170 mothers who were visited at home, 67(39.4%) of them were checked for heavy bleeding, 75(44%) of mothers were advised about their own postnatal danger sign, 19(11.2%) of mothers were measured their temperature, 11(6.5%) of mothers were measured their blood pressure at home and no mother was mentioned counseling about safe sexual practice (Table 3).

**Table 3:** Contents of maternal PNC provided by HEWs in rural Tigray, Northern Ethiopia, 2018

Postnatal contents	Frequency (N/%)
**The proportion of mothers:**	
Underwent body examination	37 (21.8%)
Checked heavy bleeding	67 (39.4%)
Measured their body Temperature	19 (11.2%)
Measured their Blood pressure	11 (6.5%)
Counseled about personal hygiene	48 (28.2%)
Counseled about family planning	34 (20%)
Checked about TT immunization	6 (3.5%)
Counseled about breastfeeding	54 (31.8%)
Counseled about own feeding	57 (33.5%)
Checked for Iron intake	4 (2.4%)

(Fig. **4**) Showed newborn contents mentioned by the mothers. A general assessment of newborns was done for 44(25.9%), newborn weighed 24(14.1%), checked their cord 70(41.2%), and 26(15.3%) newborns were measured their temperature.

**Fig. 4:**
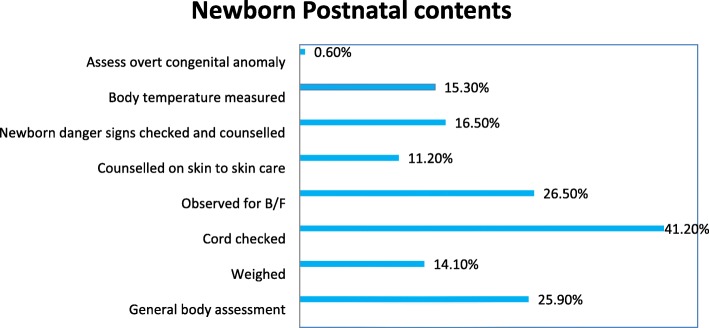
Type of PNHV contents delivered to newborns, in rural Tigray, Northern Ethiopia

### Factors associated with PNHV receipt

We calculated the adjusted odds ratio of maternal characteristics associated with the PNHV receipt. The results revealed that, mothers who received at least one home visit during pregnancy by HEWs (AOR, 7.49; CI 3.55–15.80), participated in pregnant women forum (AOR, 3.16; CI 1.67–5.99), notified their birth (AOR, 6.16; CI 3.50–10.84) and those members of community health insurance (AOR, 1.87; CI 1.13–3.10) had statistically significant positive association with postnatal home visits by health extension workers within 3 days after delivery. Educational status, the status of mothers on model family, time taken to travel health post, and availability of HEW’s cell phone at home did not show any statistically significant association with postnatal home visits (Table 4).

**Table 4:** Association of participants’ characteristics with postnatal home visit receipt, rural Tigray, northern Ethiopia, 2018 (N = 705)

Characteristics of respondents	Received PNHVs		Crude Odds Ratio		Adjusted Odds Ratio	
	Yes (%)	No (%)	Odds ratio	95% CI	Odds ratio	95% CI
Educational status of the mother						
No education	34 (12.0)	250 (88.0)	1.00		1.00	
Primary Education	31 (12.4)	219 (87.6)	1.04	0.62–1.75	0.86	0.48–1.57
Secondary education. and above	37 (21.6)	134 (78.4)	2.03	1.22–3.38	1.56	0.85–2.87
Model household status						
Did not hear and/not model family	59 (12.7)	407 (87.3)	1.00		1.00	
Yes/certified or non-certified model family	20 (29.0)	49 (71.0)	2.82	1.56–5.07	1.24	0.61–2.52
Working towards model family	23 (13.5)	147 (86.5)	1.08	0.64–1.81	0.73	0.40–1.33
ANC visit by HEW (at least1)						
No	9 (3.1)	278 (96.9)	1.00		1.00	
Yes	93 (22.2)	325 (77.8)	8.84	4.38–17.85	7.49^*^	3.55–15.80
HEWs cell phone available						
No	34 (8.9)	346 (91.1)	1.00		1.00	
Yes	68 (20.9)	257 (79.10	2.69	1.73–4.19	1.31	0.78–2.21
Participation in Pregnant women forum						
No	15 (4.7)	306 (95.3)	1.00		1.00	
Yes	87 (22.7)	297 (77.3)	5.98	3.38–10.58	3.16^*^	1.67–5.99
Community health insurance membership						
No	51 (10.7)	425 (89.3)	1.00		1.00	
Yes	51 (22.3)	178 (77.7)	2.39	1.56–3.66	1.87^*^	1.13–3.10
Birth notification						
No	19 (4.7)	387 (95.3)	1.00		1.00	
Yes	83 (27.8)	216 (72.2)	7.83	4.63–13.24	6.16^*^	3.50–10.84
Time taken to Health post						
< 30 min	81 (16.2)	420 (83.8)	1.00			
30–1 h	16 (10)	144 (90)	0.58	0.33–1.02	0.73	0.38–1.39
> 1 h	5 (11.4)	39 (88.6)	0.66	0.25–1.74	0.92	0.30–2.81

## Discussion

This study addresses an important but relatively neglected area of maternal and newborn care in northern Ethiopia. Almost all studies in northern Ethiopia have focused on facility postnatal care utilization and associated factors; PNHVs by HEWs were not assessed.

Our study showed a low coverage, 102(14.5%) mothers and newborns received PNHVs within 3 days after delivery. In our study, throughout the postnatal period, 170 (24.1%) mothers and their newborns received PNHVs by the HEWs. Full contents of PNHV both for the mother and newborn were not addressed by the HEWs. Home visits during pregnancy by HEWs, participated in pregnant women forum, birth notification to HEWs and being members of community health insurance had a statistically significant association with receipt of postnatal home visits.

Evidences showed that early postnatal home visits by community health workers reduce maternal and newborn morbidity and mortality, especially at higher coverage [14]. However, our finding showed that the percentage of mothers and neonates who received PNHV within 3 days was 102(14.5%). This is consistent with the studies on the contact coverage of postnatal visits within 48 h of birth following home births in Africa and Asia [19, 25]. Previous studies also demonstrated that the coverage of PNHVs was low with only 4 countries having over 50% [26]. Our finding is higher than a study conducted on newborn care practices at home and in health facilities in Ethiopia and Malawi [27, 28]. However, it is very low compared to other studies done in Uganda, Nepal, Bangladesh, and Ghana [20, 29, 30]. This might be due to HEWs were engaged in different activities and loaded because of increased population and multiple other competing responsibilities [31, 32].

It is known that timely and quality delivery of maternal and newborn interventions at high coverage can reduce maternal and newborn complications. However, the contents of postnatal care provided by HEWs during PNHVs to the mother and newborn, in general, were low in this study compared to previous studies [29]. This could be due to little attention given towards postnatal services by HEWs, and government officials; as evidenced from our finding that showed 418(59.3%) mothers received at least one ANC visit at home.

The findings regarding the association of home visits during pregnancy and PNHVs were mixed [33]. In this study, HEWs household visits during pregnancy had a significant positive association with receipt of PNHVs by the mothers and their newborns. ANC offers women the opportunity to access health information and to appreciate the importance of PNC. Similar findings were reported from different studies [34–36]. This might be due to frequent contact during pregnancy and forming close relationships with the mothers so that they can notify them at the delivery or in the postnatal period. Other reasons might be the HEWs were aware of the expected date of delivery (EDD) and could contact through community linkages. Thus, rigorous efforts by HEWs are necessary to visit pregnant women at home.

Early visits were also more likely if a mother had notified the HEW about the birth. Similar findings were noticed when CHWs are notified of the birth early, they are much more likely to visit home after delivery. Previous studies also have shown that mothers who have notification of their birth increase the maternal and newborn health service utilization [34].

Consistent with previous studies, Participation in the pregnant women forum was associated with receipt of postnatal home visits by HEWs. Thus, it is an opportunity to receive a continuum of care from pre-facility, facility and post facility [34].

Mothers that were members of community health insurance were more likely to receive PNHVs.

Even though, a paucity of information on the association of this variable with PNHVs found, evidences showed that maternal postnatal visit is more likely if the mother is a member of community health insurance [8]. This might also although, a postnatal home visit is for free, the need for health-seeking of the community and communication and linkage with service providers increased. Another possible reason could be mothers who received postnatal home visits might be with birth-related complications.

Therefore, in addition to pregnancy visits, efforts should make for the mothers to enroll in the community-based health insurance.

As a limitation, it is difficult to remember the accurate contents provided by health extension workers during the postnatal period because of the recall bias. The information relies on mothers’ response which was not supported by postnatal checklist. In this study, though, perceived economic status was used, the household wealth status was not assessed as a factor which may affect PNHVs.

## Conclusion

Our findings show that the coverage of postnatal home visits by HEWs remains low in rural districts of Northern Ethiopia. The factors associated with PNHVs include a home visit by HEWs during pregnancy, participation in the pregnant women forum, birth notification and, being a member of community health insurance. Existing health systems should consider interventions that improve pregnancy and birth notification strategies and more efforts should be made in improving community-based participation and linkages with community health workers.

## Data Availability

The datasets used and/or analyzed during the current study will be available from the corresponding author on reasonable request.

## Abbreviations

ANC: Antenatal Care
AOR: Adjusted Odds Ratio
BSc: Bachelor of Science
CHWs: Community Health Workers
CI: Confidence Interval
CV: Coefficient of Variance
EDD: Expected Date of Delivery
EDHS: Ethiopia Demographic Health Survey
EMDHS: Ethiopia Mini Demographic Health Survey
HEWs: Health Extension Workers
IRB: Institutional Review Board
L10K: Last Ten Kilometer
OR: Odds Ratio
PNC: Postnatal Care
PNHVs: Postnatal Home Visits
Roh: Rate of Homogeneity
SD: Standard Deviation
SPSS: Statistical Packages for Social Science
UNICEF: The United Nations International Children’s Emergency Fund
WDG: Women Development Groups
WHO: World Health Organization
